# A Clinical Case of Treatment-Emergent *Acinetobacter baumannii* Non-Susceptibility to Sulbactam-Durlobactam

**DOI:** 10.1093/cid/ciag436

**Published:** 2026-07-14

**Authors:** Sima L. Sharara, Lorena Diaz, Tomefa E. Asempa, Jose R.W. Martinez, Nicolas Canales, Yehudit Bergman, Shannon G. Murphy, Sara M. Karaba, Andrew E. Clark, Daniel V. Zurawski, Sara E. Cosgrove, Patricia J. Simner, Jose M. Munita, Pranita D. Tamma

**Affiliations:** 1 Johns Hopkins University, Baltimore, Maryland, USA;; 2 Universidad del Desarrollo, Santiago, Chile;; 3 Center for Anti-Infective Research and Development, Hartford Hospital, Hartford, Connecticut, USA;; 4 University of Pennsylvania, Philadelphia, Pennsylvania, USA;; 5 Mayo Clinic, Rochester, Minnesota, USA

**Keywords:** CRAB, sulbactam-durlobactam, antimicrobial resistance, PBP, cefiderocol

## Abstract

We report an eightfold increase in sulbactam-durlobactam MICs in a carbapenem-resistant *Acinetobacter baumannii* cerebrospinal fluid isolate recovered after 14 days of sulbactam-durlobactam plus meropenem therapy. Treatment-emergent mutations in the AdeFGH efflux system were identified, accompanied by a 166-fold increase in *adeG* expression. Efflux pump inhibition restored sulbactam-durlobactam susceptibility.

## INTRODUCTION

Carbapenem-resistant *Acinetobacter baumannii* (CRAB) are among the most challenging nosocomial pathogens due to their ability to persist in healthcare environments, evade host immune defenses, and acquire diverse antimicrobial resistance (AMR) determinants^1^. Historically, CRAB infections were managed with combination regimens composed of antibiotics with limited in vitro activity, with mortality exceeding 40%^1^. More recently, sulbactam combined with the β-lactamase inhibitor durlobactam (i.e., SUL-DUR) has become clinically available. Favorable trial results have established SUL-DUR, administered with imipenem-cilastatin (henceforth, imipenem), as a preferred therapy for CRAB infections^2,3^.

The combination of sulbactam and a carbapenem (i.e., imipenem or meropenem) demonstrates enhanced bactericidal activity, likely attributable to nonoverlapping penicillin binding protein (PBP) targets: sulbactam binds PBPs 1a/b and 3, whereas carbapenems target PBP2^4,5^. However, sulbactam is susceptible to hydrolysis by β-lactamases commonly produced by CRAB, including class A enzymes (e.g., TEM-1) and class C *Acinetobacter*-derived cephalosporinases (ADCs)^1^. In addition, both sulbactam and carbapenems are hydrolyzed by class D carbapenemases (e.g., OXA-23), frequently produced by CRAB isolates^1^. Durlobactam inhibits Class A, C, and D enzymes - notably not Class B metallo-β-lactamase (MBL) enzymes (e.g., NDM) - efficiently protecting sulbactam and carbapenems from substantial enzymatic degradation^6–8^.

Mechanisms underlying acquired SUL-DUR resistance during therapy for CRAB infections remain incompletely characterized but potentially involve amino acid changes in PBPs that reduce sulbactam affinity, along with upregulation of efflux pumps for which sulbactam and/or durlobactam are substrates^6–8^. We describe an invasive CRAB infection in which SUL-DUR minimum inhibitory concentrations (MICs) increased 8-fold following 14-days of SUL-DUR therapy. Genomic analysis and expression studies elucidated putative mechanisms of resistance.

## METHODS

### Clinical case.

A 38-year-old man with cerebral palsy complicated by hydrocephalus and longstanding ventriculoperitoneal (VP) and ventriculoarterial (VA) shunts developed ventriculitis caused by *Enterococcus raffinosus*, leading to removal of the VA shunt and proximal VP shunt, followed by placement of a new VA shunt. Two days after shunt replacement, he developed fevers and hypotension. Cerebrospinal fluid (CSF) and blood cultures grew *A. baumannii* (Day 1). High-dose ampicillin-sulbactam and meropenem were initiated; however, antimicrobial susceptibility testing (AST) demonstrated resistance to both agents. Subsequent testing showed susceptibility to SUL-DUR (20 mm) and cefiderocol (19 mm) by disk diffusion^9^. On Day 4, antibiotics were transitioned to SUL-DUR (2 grams intravenously [IV] every 6 hours, infused over 3 hours) in combination with meropenem (2 grams IV every 8 hours, infused over 3 hours). Symptoms resolved and both agents were administered for 14 days.

Soon after completing therapy, the patient re-presented with fever and emesis. Computed tomography revealed a 6.5-cm fluid collection surrounding a residual peritoneal catheter from the VP shunt that remained in situ since the initial CRAB infection. Peritoneal fluid cultures grew CRAB. SUL-DUR and meropenem were reinitiated. All remaining hardware was removed and the intra-abdominal fluid collection was drained. Subsequent disk diffusion testing demonstrated reduced susceptibility to SUL-DUR (13 mm) but continued susceptibility to cefiderocol (19 mm).

In response, treatment was modified to cefiderocol monotherapy after Day 5 of the recurrent infection (2 g IV every 8 hours, administered over 3 hours) for 4 weeks. The patient achieved complete clinical recovery.

### Antimicrobial Susceptibility Testing.

Index and subsequent isolates underwent genus and species identification using MALDI TOF (Bruker Daltonics Inc.) and AST testing using BD Phoenix^™^ (Becton Dickinson). After meropenem resistance was identified, disk diffusion was used to determine susceptibility to SUL-DUR (10μg/10μg) and cefiderocol (30 μg) (both, Hardy Diagnostics). AST for both isolates was subsequently confirmed using reference broth microdilution (BMD) for the following agents: ampicillin-sulbactam, cefiderocol, colistin, imipenem, meropenem, minocycline, and SUL-DUR. Testing was performed in triplicate to derive modal MICs. Quality control and susceptibility categorization followed the Clinical and Laboratory Standards Institute (CLSI) M100 document^9^.

### Genomic Analysis.

The index isolate was used as the reference genome for the subsequent isolate to investigate genomic changes that may have contributed to increased SUL-DUR MICs. Isolates were sequenced using short- and long-read technologies to generate hybrid assemblies. Additional details on sequencing methodology and analysis are described in the Supplemental Material.

## RESULTS

### Phenotypic Changes Following Therapy.

Following 14 days of SUL-DUR therapy, the SUL-DUR MIC increased from 1/4 μg/mL (susceptible) in the index CRAB isolate to 8/4 μg/mL (intermediate) in the subsequent isolate. Susceptibility to other tested antibiotic agents remained unchanged (Table 1).

### Genomic Correlates of Reduced Susceptibility.

Sequencing confirmed both isolates as *A. baumannii* sensu stricto, belonging to sequence type 2. They differed by 33 SNPs at the core genome level, suggestive of within-host evolution rather than reinfection with a distinct CRAB strain. Both isolates harbored the same β-lactamase genes, including *bla*_OXA-23_ (two copies each), *bla*_OXA-66_, and *bla*_ADC-73_. Furthermore, identical substitutions were identified in PBP1A (I623V), PBP1B (I110V, P112S, V726M), and PBP3 (A515V), indicating that alterations in canonical sulbactam targets were unlikely to explain the increase in SUL-DUR MIC.

Comparative genomic analysis identified two nonsynonymous mutations present exclusively in the subsequent CRAB isolate: an A247T substitution in AdeH and R327C substitution in AdeL. AdeH constitutes the outer membrane channel component of the AdeFGH multidrug efflux pump system in *A. baumannii*, whereas AdeL functions as the transcriptional regulator (i.e., repressor) controlling expression of this efflux pump^10^. No mutations were detected in other major RND efflux systems, including AdeABC and AdeIJK.

### Efflux-Mediated Mechanism of Resistance.

To assess the potential contribution of efflux-mediated resistance, sulbactam, durlobactam, and SUL-DUR MICs were determined in triplicate by BMD in the presence and absence of 50 μg/mL of PAβN, a nonspecific efflux pump inhibitor, (Supplemental Material). In the presence of PAβN, the SUL-DUR MIC of the subsequent isolate decreased from 8/4 to 0.5/4 μg/mL, while the sulbactam MIC decreased from 32 to 2 μg/mL. The durlobactam MIC was unchanged at 32 μg/mL (Supplemental Table 1). The 16-fold reduction in sulbactam MICs in the presence of PaβN suggests that reduced SUL-DUR susceptibility in the subsequent isolate was primarily driven by increased efflux of sulbactam.

Although restoration of susceptibility in the presence of PAβN supports a role for active efflux, these findings are not specific to AdeFGH, as PAβN is also expected to inhibit AdeABC and AdeIJK. Therefore, RT-qPCR was performed to compare expression of *adeG*, a component of the AdeFGH efflux system, between the isolates (Supplemental Material). Based on results of two independent biological replicates performed in triplicate, expression of *adeG* was 165.6-fold higher in the subsequent isolate; expression of the housekeeping gene *rpoB* remained stable (Supplemental Table 2). Together, these findings implicate marked AdeFGH upregulation as the likely driver of reduced SUL-DUR susceptibility during therapy.

## DISCUSSION

Reduced susceptibility to SUL-DUR emerged in a CRAB isolate following 14 days of therapy and was most likely attributable to treatment-emergent alterations in the AdeFGH efflux system. The restoration of SUL-DUR susceptibility following efflux pump inhibition supports enhanced AdeFGH activity as a plausible mechanism underlying the observed MIC increase. Consistent with this hypothesis, significantly increased *adeG* expression in the subsequent isolate implicates AdeFGH-mediated efflux in the development of reduced SUL-DUR susceptibility.

SUL-DUR nonsusceptibility remains uncommon in the United States, with over 95% susceptibility among CRAB isolates^11,12^. This reflects the low prevalence of MBL-producing CRAB, as durlobactam lacks activity against class B carbapenemases^6–8^. In contrast to the United States, MBL-producing CRAB account for approximately 42%, 36%, and 18% of isolates in the Middle East, Africa, and South and Southeast Asia, respectively^13^.

Beyond β-lactamase-mediated resistance, alterations in PBP targets and enhanced efflux activity also modulate SUL-DUR susceptibility and may become more relevant with expanded use. PBP3 substitutions appear particularly important: among 59 SUL-DUR-nonsusceptible isolates without prior drug exposure, 81% harbored PBP3 mutations^14^. Similarly, all eight index SUL-DUR-nonsusceptible isolates (5% [8/175]) in a clinical trial contained substitutions at or near the PBP3 active site (T526S [n=3], A515V [n=3], G523V [n=1], and N377Y plus T526S [n=1])^7^. Complementary targeting of PBP2 by carbapenems may partially mitigate the impact of such alterations, as suggested by in vitro pharmacodynamic studies demonstrating restored bactericidal activity with combined PBP inhibition^4,5^.

Encouragingly, treatment-emergent SUL-DUR resistance is uncommon. In the same clinical trial, resistance developed in fewer than 1% of patients receiving SUL-DUR therapy^2^. Nevertheless, additional resistance mechanisms may emerge during SUL-DUR exposure. The AdeABC efflux system mediates sulbactam extrusion^15^, while AdeIJK contributes to intrinsic β-lactam resistance and may also transport durlobactam^6,15^. In the present case, AdeABC and AdeIJK were unchanged; resistance coincided with mutations in AdeFGH, a broad-spectrum multidrug efflux system. Although the contribution of AdeFGH to sulbactam or durlobactam resistance has not previously been described, the marked reduction in sulbactam MICs following efflux inhibition along with increased *adeG* expression in the subsequent isolate supports a functional role for AdeFGH-mediated extrusion. Consistent with prior reports, target or efflux-mediated resistance typically results in moderate SUL-DUR MIC increases (8–16 μg/mL), whereas NDM-mediated resistance produces substantially higher MICs^6,14^; a distinction that may help guide clinical interpretation of susceptibility results when genomic data are not available.

Collectively, this case implicates mutation-driven activation of the AdeFGH efflux system as a plausible mechanism of treatment-emergent SUL-DUR resistance. These findings underscore the importance of repeat susceptibility testing and genomic surveillance in patients with persistent or recurrent CRAB infections receiving SUL-DUR therapy.

## Supplementary Material

Supplemental Data

## Figures and Tables

**Table 1:** Modal results of triplicate reference broth microdilution testing for index and subsequent carbapenem-resistant *Acinetobacter baumannii* (CRAB) isolates obtained after 14 days of sulbactam-durlobactam therapy

Isolate	Cefiderocol MIC	Colistin MIC	Imipenem MIC	Meropenem MIC	Minocycline MIC	Sulbactam MIC	Sulbactam-durlobactam MIC
Index	2 μg/mL (S)	2 μg/mL (I)	32 μg/mL (R)	64 μg/mL (R)	4 μg/mL (R)	16 μg/mL (R)	1/4 μg/mL (S)
Subsequent	2 μg/mL (S)	2 μg/mL (I)	32 μg/mL (R)	64 μg/mL (R)	4 μg/mL (R)	32 μg/mL (R)	8/4 μg/mL (I)

S = susceptible, I = intermediate, R = resistant

MIC testing limited to agents considered preferred or alternative components of combination therapy for CRAB infections, according to the 2026 IDSA AMR Treatment Guidance^3^

## Data Availability

Whole-genome sequencing reads generated in this study are publicly available in the National Center for Biotechnology Information Sequence Read Archive under BioProject accession numbers PRJNA56497060 (index isolate) and PRJNA56497061 (subsequent isolate).
