# Supplementary material for: A Clinical Case of Treatment-Emergent Acinetobacter baumannii Non-Susceptibility to Sulbactam-Durlobactam
Source: Clin Infect Dis. Author manuscript; Available in PMC 2026 Sep 16. (PMC13579459; doi:10.1093/cid/ciag436)
Supplement: Supplementary file 1 [file NIHMS2209357-supplement-Supplemental_Data.docx]

**Supplemental Materials**

**Whole Genome Sequencing and Bioinformatic Analysis.** The index carbapenem-resistant *Acinetobacter baumannii* (CRAB) isolate was used as the reference genome for the subsequent isolate to investigate genomic changes that may have contributed to increased sulbactam-durlobactam (SUL-DUR) minimum inhibitory concentrations (MICs). Mutations previously implicated in sulbactam or durlobactam resistance were investigated by a targeted literature review^1-5^. These included changes potentially altering sulbactam affinity to PBP1A (e.g., A219T), PBP1B (e.g., P112S), or PBP3 (e.g., A515V); changes potentially affecting durlobactam affinity to PBP2 (e.g., P665A); or mutations modifying the function of key intrinsic efflux pump systems (e.g., AdeIJK), including regulatory genes (e.g., *adeN*). Genomic DNA from the index and subsequent CRAB isolates was extracted using the DNeasy PowerSoil Pro Kit (QIAGEN, Germantown, MD, USA) and sequenced using both short- and long-read technologies.

Short-read sequencing was performed on the Illumina NextSeq 2000 platform, generating 150 bp paired-end reads with a target coverage depth exceeding 30x. Libraries were prepared using Illumina DNA Prep kit. Long-read sequencing was performed using the Oxford Nanopore GridION platform on Flow Cell (FLO-MIN114), generating long reads to facilitate resolution of repetitive regions and structural variation. Libraries were prepared with Rapid Barcoding Kit V14 (SQK-RBK114.96). Raw reads quality from both platforms was assessed using FastQC v0.11.9 and MultiQC v1.10.1. Illumina reads were trimmed and filtered using FastP (v0.23.4), excluding bases with a Phred quality score below 30. A *de novo* assembly strategy was performed with SPAdes (v3.15.5). Nanopore reads were base-called using Guppy (v6.5.7). Reads shorter than 1 kb using NANOQ (v0.5.0) or with mean quality below Q7 were removed. Adapter sequences were removed with Porechop-ABI (v0.10.0). *De novo* assembly was performed using Flye (v2.9.3) and polished for correcting base-call errors with Medaka (v1.11.3). Assembly quality metrics, including N50, total assembly length, and completeness, were evaluated using QUAST v5.2.0. Genome annotation was performed using Bakta (v1.8.2), and multilocus sequence type was assigned using the MLST tool (v2.23.0) with the PubMLST database. Assembled contigs were screened for acquired AMR determinants and point mutations associated with AMR using AMRFinderPlus, applying thresholds of 100% nucleotide identity and ≥80% gene coverage. To investigate AMR markers not available through AMRFinderPlus, the index and subsequent isolate genomes were aligned to a reference genome (*A. baumannii* ATCC19606, Genbank accession: GCA_009035845.1). Additionally, the presence of novel single nucleotide polymorphisms potentially associated with increased SUL-DUR MICs were assessed with Snippy (v4.6.0) using the index genome as the reference.

**Non-Specific Efflux Pump Inhibition.** As both sulbactam and durlobactam are substrates of intrinsic efflux systems, additional testing was conducted to investigate whether mutations in efflux systems may have contributed to the emergence of SUL-DUR resistance. Sulbactam, durlobactam, and SUL-DUR MICs were determined in triplicate by BMD in the presence and absence of 50 µg/mL of phenylalanine-arginine-β-naphthylamide (PAβN), a nonspecific efflux pump inhibitor^6^. All experiments included *A. baumannii* NCTC 13304 as a quality control strain. Results are displayed in **Supplemental Table 1**.

**RT-qPCR Analysis.** Reverse transcription quantitative PCR (RT-qPCR) was performed to assess expression of the AdeFGH efflux system through quantification of *adeG* transcript levels. AdeFGH is a resistance-nodulation-division (RND) family multidrug efflux pump in *A. baumannii* composed of *adeF*, encoding the membrane fusion protein; *adeG*, encoding the RND transporter; and *adeH*, encoding the outer membrane channel protein. The *adeG* gene was selected as the target gene because it encodes the transporter component of the tripartite efflux system. The *rpoB* gene, encoding the RNA polymerase β-subunit, served as the endogenous reference gene. Relative *adeG* expression in the subsequent CRAB isolate was quantified relative to the index isolate. Primer and probe sequences were designed using the *A. baumannii* AB2016 reference genome (GenBank accession no. CP199977.1).

Bacterial cultures were grown to mid-logarithmic phase and immediately stabilized with RNAprotect Bacteria Reagent (Qiagen, Hilden, Germany), according to manufacturer’s instructions. Total RNA was extracted using the RNeasy Protect Bacteria Mini Kit (Qiagen, Hilden, Germany) and treated with TURBO DNase (Thermo Fisher Scientific, Waltham, MA, USA) to remove residual genomic DNA. RNA concentration and purity were assessed using a NanoDrop OneC microvolume UV-Vis spectrophotometer (Thermo Fisher Scientific, Waltham, MA, USA). The following primers and hydrolysis probes were used:

***adeG***

Forward primer (*adeG*-F): 5′-AGGCTTAACAGGGCAATTCT-3′

Reverse primer (*adeG*-R): 5′-CCAAAGCAGGAGATAGGGTAAG-3′

Probe (*adeG*-probe): 5′-/56-FAM/TGCCATGAC/ZEN/CATTGCCATTTCAACG/3IABkFQ/-3′

***rpoB***

Forward primer (*rpoB*-F): 5′-GCTTCAGGAACATCGTTTGGT-3′

Reverse primer (*rpoB*-R): 5′-TGCATCAGCTTGTCGTTGAT-3′

Probe (*rpoB*-probe): 5′-/56-FAM/ACCAGCTTTGGTGAAGGTGGTGTTG/ZEN/3IABkFQ/-3′

RT-qPCR was performed using TaqPath™ 1-Step RT-qPCR Master Mix, CG (Thermo Fisher Scientific, Waltham, MA, USA) on a QuantStudio™ 5 Real-Time PCR System (Applied Biosystems, Foster City, CA, USA). Reverse transcription and PCR amplification were performed in a single reaction. Each 20-μL reaction contained 5 μL of 4× TaqPath Master Mix, gene-specific primers (final concentration, 900 nM each), probe (final concentration, 250 nM), and 35 ng of total RNA template. All assays were performed in technical triplicate for two independent biological replicates generated from separate nucleic acid extractions. No-template controls and no-reverse-transcriptase controls were included on each plate to monitor contamination and confirm the absence of genomic DNA carryover.

Thermal cycling conditions consisted of uracil-N-glycosylase activation at 25°C for 2 minutes, reverse transcription at 50°C for 15 minutes, polymerase activation and initial denaturation at 95°C for 2 minutes, followed by 40 cycles of 95°C for 3 seconds and 60°C for 30 seconds. Standard curves generated from 10-fold serial dilutions of RNA were used to determine amplification efficiencies for both *adeG* and *rpoB*. Relative gene expression was calculated using the efficiency-corrected Pfaffl method^7^, which incorporates gene-specific amplification efficiencies and cycle threshold differences between target and reference genes. A summary of the RT-qPCR results is available in **Supplemental Table 2**.

| **Supplemental Table 1**. Effect of efflux pump inhibition with phenylalanine-arginine-β-naphthylamide (PAβN) on sulbactam, durlobactam, and sulbactam-durlobactam minimum inhibitory concentrations (MICs) in index and subsequent carbapenem-resistant *Acinetobacter baumannii* isolates | | | | | | | |
| --- | --- | --- | --- | --- | --- | --- | --- |
|  |  | Sulbactam MIC (µg/mL) | | Durlobactam MIC (µg/mL) | | SUL-DUR MIC (µg/mL) | |
| Isolate | Run | CAMHB | CAMHB + PABN 50 mg/L | CAMHB | CAMHB + PABN 50 mg/L | CAMHB | CAMHB + PABN 50 mg/L |
| Index | a | 8 | 1 | 32 | 32 | 1 | 0.5 |
| Index | b | 16 | 1 | 32 | 32 | 1 | 0.5 |
| Index | c | 16 | 1 | 32 | 32 | 1 | 0.5 |
| Subsequent | a | 32 | 2 | 32 | 32 | 8 | 0.5 |
| Subsequent | b | 32 | 2 | 32 | 16 | 16 | 0.25 |
| Subsequent | c | 32 | 2 | 64 | 32 | 8 | 0.5 |

| **Supplemental Table 2.** Summary of reverse transcription quantitative PCR results comparing *adeG* and *rpoB* expression between index and subsequent carbapenem-resistant *Acinetobacter baumannii* isolates | | |
| --- | --- | --- |
| **Parameter** | **Index isolate** | **Subsequent isolate** |
| Mean *adeG* CT | 24.131 ± 0.179 | 16.571 ± 0.078 |
| Mean *rpoB* CT | 16.571 ± 0.252 | 16.609 ± 0.397 |
| ΔCT (biological replicate a) | 7.884 | 0.422 |
| ΔCT (biological replicate b) | 7.073 | -0.497 |
| Mean ΔCT | 7.559 ± 0.452 | -0.038 ± 0.458 |
| Fold Change | 1.00 (reference) | 165.6 |

**References**

1.	Moussa SH, Shapiro AB, McLeod SM, et al. Molecular drivers of resistance to sulbactam-durlobactam in contemporary clinical isolates of Acinetobacter baumannii. Antimicrob Agents Chemother. Nov 15 2023;67(11):e0066523. doi:10.1128/aac.00665-23.

2.	McLeod SM, Shapiro AB, Moussa SH, et al. Frequency and Mechanism of Spontaneous Resistance to Sulbactam Combined with the Novel beta-Lactamase Inhibitor ETX2514 in Clinical Isolates of Acinetobacter baumannii. Antimicrob Agents Chemother. Feb 2018;62(2)doi:10.1128/AAC.01576-17.

3.	Miller AA, Moussa SH, McLeod SM. Characterization of Acinetobacter baumannii-calcoaceticus complex isolates and microbiological outcome for patients treated with sulbactam-durlobactam in a phase 3 trial (ATTACK). Antimicrob Agents Chemother. May 2 2024;68(5):e0169823. doi:10.1128/aac.01698-23.13.

4.	Principe L, Di Bella S, Conti J, et al. Acinetobacter baumannii Resistance to Sulbactam/Durlobactam: A Systematic Review. *Antibiotics (Basel)*. Dec 10 2022;11(12)doi:10.3390/antibiotics11121793.

5.	Xu Q, Liu X, Liu H, et al. In vitro activity and resistance mechanisms of sulbactam/durlobactam against Acinetobacter baumannii clinical isolates in China (2019-2020). *Clin Microbiol Infect*. Jan 2026;32(1):102–109. doi:10.1016/j.cmi.2025.09.016.

6.	Lamers RP, Cavallari JF, Burrows LL. The efflux inhibitor phenylalanine-arginine beta-naphthylamide (PAbetaN) permeabilizes the outer membrane of gram-negative bacteria. *PLoS One*. 2013;8(3):e60666. doi:10.1371/journal.pone.0060666.

7.	Pfaffl MW. A new mathematical model for relative quantification in real-time RT-PCR. Nucleic Acids Res. 2001;29(9):e45. doi:10.1093/nar/29.9.e45.
